# Supplementary material for: Economical management of virtual power plant source-network-load-storage in the context of electricity-carbon market
Source: PLoS One. 2026 Jan 7;21(1):e0338321. doi: 10.1371/journal.pone.0338321 (PMC12779080; doi:10.1371/journal.pone.0338321)
Supplement: Attachment — Submitted filename: Response to Reviewers.docx [file pone.0338321.s001.docx]

**Authors’ response to the review of paper PONE-D-25-05045**

**Paper title:**	Economical Management of Virtual Power Plant Source-network-load-storage in the Context of Electricity-Carbon Market

**Authors:**	Panhong Zhang and Yilu Zheng

**Manuscript number:	PONE-D-25-05045**

**Submission 1:**	Jan 29 2025

**Revision 1 request:**	Mar 31 2025

**Submission 2:**	May 15 2025

Editor’s Comments

*Thank you for submitting your manuscript to* *PLOS ONE. After careful consideration, we feel that it has merit but does not fully meet PLOS ONE’s publication criteria as it currently stands. Therefore, we invite you to submit a revised version of the manuscript that addresses the points raised during the review process.*

The Reviewers have meticulously examined our manuscript and presented us with a very constructive set of comments and requests, which we believe have improved the strength of our manuscript and the presentation of our work significantly. In the following pages, we address each and every comment of all the Reviewers. Each comment is marked by the Reviewer’s and comment’s index and includes the Reviewer’s text in the original form and content for convenience of reference. Each section discusses one comment, and refers to the particular point of our revised manuscript that has been amended to accommodate this comment.

We have endeavoured to take all the remarks and requests of the Reviewers strongly into account, and have addressed all comments analytically and very carefully, no matter how minor or major they were. We hope our revision is now up to the standards of **PLOS ONE** and if requested, we would be glad to address any further issues in subsequent revisions.

Additional Editor Comments

*The introduction section needs considerable effort (concise and brief). The problem being investigated should be described clearly, but before that, the field of research should be made clearer. Furthermore, briefly describe the major contributions in bullet form, just before the organization paragraph.*

*The introduction should lead the way throughout the paper. In addition, the benefits coming from this paper*

*should be made clearer in the introduction and throughout the paper.*

*I suggest summarizing the related works into a table with respect to their characteristics. The authors should put their proposal into this table for easy comparison.*

*This will make it clearer to readers, and they will be able to see what was missing in the literature and how this is addressed in this paper. Proofread the article to ensure appropriate use of English grammar, tenses, and punctuation. Longer sentences should be broken out into smaller ones. There are also some linguistic issues that should be corrected. The use of article "the" is redundant and somewhere missing..*

First of all, we have restructured the introduction to start with a concise and focused overview of the research domain, particularly emphasizing the challenges of energy management in VPPs under the dual-carbon target and electricity-carbon market background. We have updated our references with the latest researches, including the suggested references from reviewers and other references:

[11]	Alsokhiry, F.; Annuk, A.; Mohamed, M.A.; Marinho, M. An innovative cloud-fog-based smart grid scheme for efficient resource utilization. Sensors. **2023,** 23, 1752.

[12]	Li, P.; Zheng M. Multi-objective optimal operation of hybrid AC/DC microgrid considering source-network-load coordination, Modern Power Systems and Clean Energy, **2023**, 7, 1229.

[16]	Wang, Q.; Leng, Z.; Tan, H.; Mohamed A. Mohamed. Optimal scheduling of virtual power plants with reversible solid oxide cells in the electricity market, Renewable Energy. **2024**, 237, 121759.

[18]	He, Y.; Wu, X.; Sun, K.; Du, X.; Wang, H.; Zhao, J. Economic optimization scheduling based on load demand in microgrids considering source network load storage. Electronics. **2023**, 12, 2721.

[19]	Mohamed A.; Heba M.; Mohammed A.; Mohamed Sharaf.; Ahmed T. A novel fuzzy cloud stochastic framework for energy management of renewable microgrids based on maximum deployment of electric vehicles, International Journal of Electrical Power & Energy Systems. **2021**, 129, 106845.

[25]	Liu, X. Research on bi-layer low carbon scheduling strategy for source-load collaborative optimization based on node carbon emission intensity. Energy Science & Engineering. **2024**; 12: 713-735.

Following this comment, the introduction presents a comprehensive examination of VPP source–network–load–storage co-optimization to underscore the novelty of the proposed framework. The review not only provides a thorough background based on state-of-the-art research but also critically identifies the limitations of existing approaches. Until now, extensive researches in [4]-[19] on VPP is mainly limited to parts of source–network–load–storage, including source–storage, source–load–storage, source–network, etc. Though VPP source–network–load–storage is investigated in [20]-[25], multi-timescale cloud-edge-end based the VPP source-network-load-storage in the context of electricity-carbon market has not been explored. Cloud-edge-end frameworks enhance the flexibility, resilience, and efficiency of VPPs, enabling them to better respond to fluctuations in energy supply and demand, especially in the context of a rapidly evolving electricity-carbon market. Real-time pricing (RTP) and time of use (TOU) are two typical pricing mechanisms to facilitate the VPP source-network-load-storage interactions. Though RTP and TOU pricing mechanisms drive more intelligent interactions between VPP source-network-load-storage, the VPP supply–demand behavior is generally ignored. While the end-users can receive financial benefits from the pricing mechanisms, overall system load profile cannot be flattened. These research gaps and associated challenges are further elaborated in Section 1.2:

“*1.2. Literature review*

*Until now, extensive researches on VPP is mainly limited to parts of source–network–load–storage, including source–storage, source–load–storage, source–network, etc. In the VPP source-network aspect, studies mainly focus on the coordination between distributed energy sources and the power network to enhance system stability and flexibility. In [4],* *advanced control technologies are employed to optimize the interaction between distributed generation units and the grid, improving voltage regulation, frequency support, and fault management capabilities. The complementary characteristics of various RESs are highlighted in [5], since their diverse generation profiles can compensate for each other's intermittency. In [6–7], an enhanced real-time economic scheduling algorithm is developed to improve the accuracy of output prediction for distributed energy resources. In [8], a resilient restoration method is proposed for VPP power-traffic network, which co-optimizes the deployment of mobile power sources and network reconfiguration. In [9], a multi-energy coordination framework is proposed to enhance the VPP resilience against large-scale disturbances, where consumer-side biogas-solar-wind multi-energy VPPs are topologized by network reconfiguration. In [10], the integration of multi-source coordinated scheduling within VPPs is analyzed, with particular emphasis on its performance in ancillary service markets.*

*In the VPP source-load-storage and source-network-load aspects, demand response and load shedding strategies [11] are extensively utilized to enhance system flexibility by dynamically adjusting energy consumption behaviors and patterns. In [12], a multi-objective source-network-load coordinated model is proposed and solved an improved memetic algorithm, considering the adjustment of load demand curves. To exploit the inherent similarities between RESs and load profiles, a two-layer VPP optimization framework is proposed in [13] to jointly coordinate ESS and industrial loads. In [14], an economic scheduling optimization model is developed, explicitly considering the effects of temperature variations on PV generation performance. In [15], a SOC-based optimization approach is introduced to minimize overall economic costs, system losses, and line voltage deviations through a penalty function formulation. To investigate the beneficial effect of bi-directional electric-hydrogen conversion on market trading, an optimal scheduling method is proposed in [16] for VPP with reversible solid oxide cells in the electricity market. Given the escalating complexity of dispatchable entities within VPP operations, significant attention has been directed toward enhancing dispatch efficiency [17]. In [18],* *an economic VPP scheduling model considering the load demand under the mechanism of a peak–valley tariff is proposed. To address the uncertainties associated with renewable energy integration, a novel intelligent methodology is proposed in [19] based on fuzzy cloud theory and honeybee mating algorithm.*

*In the source-network-load-storage aspect, the maximum utilization of energy resources is achieved through multiple forms of interaction between power sources, grids, loads and energy storage. In [20], a stochastic economical optimization strategy is proposed for VPPs, addressing scenarios both with and without demand response programs. Based on model predictive control, a multi-stage optimal source-network-load-storage scheduling model is proposed in [21] to ensure the safe and economic operation. In [22], a source-network-load-storage optimization model is introduced, where a point-to-point vehicle charging strategy is implemented. In [23], an integrated source-grid-load-storage monitoring platform based on the micro-grid in State Grid Shanghai Electrical Power Research Institude and blockchain technology. In [24], a novel multi-stage time scale economic dispatch scheme is proposed for VPPs, including a fuzzy optimized day ahead scheduling scheme, an intra-day scheduling scheme combined with Deep Q Network, and an adaptive optimized real-time scheduling scheme. In [25], a bi-layer low-carbon scheduling model is proposed based on node carbon emission intensity. The integration of cloud-edge-end technologies in VPPs is becoming increasingly necessary due to the growing complexity of managing distributed energy resources and ensuring grid stability. VPPs rely on real-time data processing and coordination between various sources of generation, storage, and demand, which can be geographically dispersed and highly dynamic. Together, cloud-edge-end frameworks enhance the flexibility, resilience, and efficiency of VPPs, enabling them to better respond to fluctuations in energy supply and demand, especially in the context of a rapidly evolving electricity-carbon market. However, multi-timescale cloud-edge-end based the VPP source-network-load-storage in the context of electricity-carbon market has not been explored.*

*Real-time pricing (RTP) and time of use (TOU) are two typical pricing mechanisms to facilitate the VPP source-network-load-storage interactions. While RTP reflects the actual cost of electricity based on real-time supply and demand conditions in the market, TOU is set based on predefined time periods, typically distinguishing between peak, off-peak, and mid-peak hours. Though RTP and TOU pricing mechanisms drive more intelligent interactions between VPP source-network-load-storage, the VPP supply–demand behavior is generally ignored. While the end-users can receive financial benefits from the pricing mechanisms, overall system load profile cannot be flattened.”*

Then, to provide a clearer overview of prior research and position our work effectively, we have added a new comparison table. Our method is also included in this table for direct comparison, helping to highlight the novelty and addressed gaps. Following this comment, a comparative table is provided to show the advantages of this work.

*Table 1. DIFFERENCES WITH THE PROPOSED APPROACH*

| *References* | *source-network* | *source-load-storage* | *source-network-load* | *source-network-load-storage* | *pricing* | *Cloud-edge-end* |
| --- | --- | --- | --- | --- | --- | --- |
| *[4][5][9]* | *√* |  |  |  | *TOU* |  |
| *[6][7][8]* | *√* |  |  |  | *RTP* |  |
| *[11]* |  |  | *√* |  | *TOU* | *√* |
| *[12][14]* |  |  | *√* |  | *TOU* |  |
| *[13][15][18]* |  | *√* |  |  | *TOU* |  |
| *[10][16][19]* |  | *√* |  |  | *RTP* |  |
| *[17]* |  |  | *√* |  | *RTP* |  |
| *[20]* |  |  |  | *√* | *RTP* |  |
| *[21]-[25]* |  |  |  | *√* | *TOU* |  |
| *Proposed* |  |  |  | *√* | *interactive RTP* | *√* |

Furthermore, we have revised the contribution section to more clearly and highlight the strengths of the paper. The main contributions of this paper are summarized as follows: 1) While the previous literature is mainly limited to the parts of source–network–load–storage, the inherent system availability and dispatchability from other flexible parts cannot be fully exploited. Moreover, the lack of integrated control strategies often leads to suboptimal system performance and resource utilization. This paper proposes a unified VPP source–network–load–storage management framework, which leverages cloud-edge-end collaborative technologies to achieve the real-time data processing and coordination. Through advanced communication and control mechanisms, heterogeneous energy resources can be managed in a more synchronized manner. This framework enables coordinated and flexible control of distributed generation, distribution networks, EVs, and energy storage systems, thereby reducing both system energy consumption and operational costs; 2) A two-layer cloud–edge–end-based multi-time scale economic management framework for VPP source–network–load–storage is proposed to enable dynamic and hierarchical control of the system. In the first layer, day-ahead economic scheduling of source–network–load–storage resources is performed at the cloud–edge level, ensuring optimal allocation based on forecasted data. In the second layer, a novel real-time supply-demand pricing mechanism is introduced to optimize the scheduling of EV storage and charging stations based on end-side MPC, allowing the system to adapt flexibly to real-time fluctuations. Compared to a single-layer framework, the two-layer model offers enhanced cost-effectiveness and is easier to implement, providing a scalable solution that better accommodates the growing complexity of distributed energy resources; 3) While RTP and TOU pricing mechanisms generally ignore the supply–demand behavior, an interactive real-time pricing mechanism is proposed for EV storage and charging stations. This mechanism dynamically adjusts prices based on real-time supply–demand conditions, aiming to better align economic incentives with system needs. By continuously monitoring system fluctuations, it can more effectively balance load and generation. Moreover, it encourages flexible EV charging behaviors, ultimately enhancing overall grid stability and efficiency. This has been clarified in Section 1.3:

*“1.3. Contribution and paper organization*

*Based on the cloud-edge-end collaborative technology, this paper proposes a multi-time scale VPP source-network-load-storage management framework. This paper makes the following contributions:*

*1) While the previous literature is mainly limited to the parts of source–network–load–storage, the inherent system availability and dispatchability from other flexible parts cannot be fully exploited. This paper proposes a unified VPP source–network–load–storage management framework, which leverages cloud-edge-end collaborative technologies to achieve the real-time data processing and coordination. This framework enables coordinated and flexible control of distributed generation, distribution networks, EVs, and energy storage systems, thereby reducing both system energy consumption and operational costs.*

*2) A two-layer cloud–edge–end-based multi-time scale economic management framework for VPP source–network–load–storage is proposed to enable dynamic and hierarchical control of the system. In the first layer, day-ahead economic scheduling of source–network–load–storage resources is performed at the cloud–edge level.* *In the second layer, a novel real-time supply-demand pricing mechanism is introduced to optimize the scheduling of EV storage and charging stations based on end-side model predictive control (MPC). Compared to a single-layer framework, the two-layer model offers enhanced cost-effectiveness and is easier to implement.*

*3) While RTP and TOU pricing mechanisms generally ignore the supply–demand behavior, an interactive real-time pricing mechanism is proposed for EV storage and charging stations. This mechanism dynamically adjusts prices based on real-time supply–demand conditions, aiming to better align economic incentives with system needs.*

*4) To address the non-convexity inherent in the source-network-load-storage and real-time electricity pricing model, a mixed-integer linear programming (MILP) relaxation is employed. This approach ensures the preservation of convexity and enhances the convergence performance of the optimization process.”*

Finally, the entire manuscript has been thoroughly proofread and edited for grammar, sentence structure, tense consistency, and correct usage of articles. We have broken down longer sentences into shorter, clearer ones and corrected redundant or missing use of the article “the” as noted.

Reviewer 1

This paper proposes a cloud-edge-end-based economical management of virtual power plant source-network-load-storage in the context of electricity-carbon market. The manuscript presents some significant results worthy of publication but should be revised to be more precise and accurate in the wording of the text. Some of my comments are listed as follows:

Comment 1:

*The manuscript contains several typographical as well as grammatical errors. Authors are suggested to get the manuscript proofread by a native English speaker.*

The authors sincerely thank the Reviewer for highlighting the typographical and grammatical errors. We have carefully revised the manuscript and conducted a thorough proofreading to enhance the overall language quality.

Comment 2:

*The abstract only state the experimental or calculated process of this work. The authors should be given specific result to demonstrate the research significance.*

As suggested, the Abstract section is enhanced with some numerical results added. Simulations results show that the system operating cost can be reduced by at most 2.35% with a higher flexibility of electricity market operations. This has been clarified in Abstract: *“Under the imperative of achieving dual-carbon goals, the number of distributed energy resources are gradually increasing, thereby amplifying the challenges to grid stability and power balance. Consequently, there is an urgent need to leverage the potential of source-network-load-storage for enhanced power regulation and control. This paper proposes a cloud-edge-end-based multi-time scale economical management of virtual power plant (VPP) source-network-load-storage in the context of electricity-carbon market. In the first layer, a cloud-edge scheduling approach is used to optimize the source-network-load-storage system of the VPP over a long time horizon, aiming to maximize economic benefits. In the second layer, a novel real-time pricing mechanism is employed to effectively manage and regulate the electric vehicle (EV) storage and charging stations. After obtaining the economic management parameters from the previous layer, the second layer employs a real-time scheduling approach based on end-side model predictive control (MPC), to address multi-energy supply-demand fluctuations. To achieve efficient solution, the original two-layer optimization problem is reformulated using mixed-integer linear programming (MILP). Comparative analyses have demonstrated the superior economic and practical performance of the pro-posed two-layer optimization approach. Simulation results indicate that the total operating cost of the system can be reduced by 2.35%, with a higher flexibility of electricity market operations.”*

Comment 3:

*The introduction can be improved by providing a more critical discussion of recent related literature. Discuss the shortcomings of previous work and the gaps, and how this work intends to fill those gaps. The authors should determine the novelty of the current research in the last paragraph of the introduction part.*

First of all, we have restructured the introduction to start with a concise and focused overview of the research domain, particularly emphasizing the challenges of energy management in VPPs under the dual-carbon target and electricity-carbon market background. We have updated our references with the latest researches, including the suggested references from reviewers and other references:

[11]	Alsokhiry, F.; Annuk, A.; Mohamed, M.A.; Marinho, M. An innovative cloud-fog-based smart grid scheme for efficient resource utilization. Sensors. **2023,** 23, 1752.

[12]	Li, P.; Zheng M. Multi-objective optimal operation of hybrid AC/DC microgrid considering source-network-load coordination, Modern Power Systems and Clean Energy, **2023**, 7, 1229.

[16]	Wang, Q.; Leng, Z.; Tan, H.; Mohamed A. Mohamed. Optimal scheduling of virtual power plants with reversible solid oxide cells in the electricity market, Renewable Energy. **2024**, 237, 121759.

[18]	He, Y.; Wu, X.; Sun, K.; Du, X.; Wang, H.; Zhao, J. Economic optimization scheduling based on load demand in microgrids considering source network load storage. Electronics. **2023**, 12, 2721.

[19]	Mohamed A.; Heba M.; Mohammed A.; Mohamed Sharaf.; Ahmed T. A novel fuzzy cloud stochastic framework for energy management of renewable microgrids based on maximum deployment of electric vehicles, International Journal of Electrical Power & Energy Systems. **2021**, 129, 106845.

[25]	Liu, X. Research on bi-layer low carbon scheduling strategy for source-load collaborative optimization based on node carbon emission intensity. Energy Science & Engineering. **2024**; 12: 713-735.

Following this comment, the introduction presents a comprehensive examination of VPP source–network–load–storage co-optimization to underscore the novelty of the proposed framework. The review not only provides a thorough background based on state-of-the-art research but also critically identifies the limitations of existing approaches. Until now, extensive researches in [4]-[19] on VPP is mainly limited to parts of source–network–load–storage, including source–storage, source–load–storage, source–network, etc. Though VPP source–network–load–storage is investigated in [20]-[25], multi-timescale cloud-edge-end based the VPP source-network-load-storage in the context of electricity-carbon market has not been explored. Cloud-edge-end frameworks enhance the flexibility, resilience, and efficiency of VPPs, enabling them to better respond to fluctuations in energy supply and demand, especially in the context of a rapidly evolving electricity-carbon market. Real-time pricing (RTP) and time of use (TOU) are two typical pricing mechanisms to facilitate the VPP source-network-load-storage interactions. Though RTP and TOU pricing mechanisms drive more intelligent interactions between VPP source-network-load-storage, the VPP supply–demand behavior is generally ignored. While the end-users can receive financial benefits from the pricing mechanisms, overall system load profile cannot be flattened. These research gaps and associated challenges are further elaborated in Section 1.2:

“*1.2. Literature review*

*Until now, extensive researches on VPP is mainly limited to parts of source–network–load–storage, including source–storage, source–load–storage, source–network, etc. In the VPP source-network aspect, studies mainly focus on the coordination between distributed energy sources and the power network to enhance system stability and flexibility. In [4],* *advanced control technologies are employed to optimize the interaction between distributed generation units and the grid, improving voltage regulation, frequency support, and fault management capabilities. The complementary characteristics of various RESs are highlighted in [5], since their diverse generation profiles can compensate for each other's intermittency. In [6–7], an enhanced real-time economic scheduling algorithm is developed to improve the accuracy of output prediction for distributed energy resources. In [8], a resilient restoration method is proposed for VPP power-traffic network, which co-optimizes the deployment of mobile power sources and network reconfiguration. In [9], a multi-energy coordination framework is proposed to enhance the VPP resilience against large-scale disturbances, where consumer-side biogas-solar-wind multi-energy VPPs are topologized by network reconfiguration. In [10], the integration of multi-source coordinated scheduling within VPPs is analyzed, with particular emphasis on its performance in ancillary service markets.*

*In the VPP source-load-storage and source-network-load aspects, demand response and load shedding strategies [11] are extensively utilized to enhance system flexibility by dynamically adjusting energy consumption behaviors and patterns. In [12], a multi-objective source-network-load coordinated model is proposed and solved an improved memetic algorithm, considering the adjustment of load demand curves. To exploit the inherent similarities between RESs and load profiles, a two-layer VPP optimization framework is proposed in [13] to jointly coordinate ESS and industrial loads. In [14], an economic scheduling optimization model is developed, explicitly considering the effects of temperature variations on PV generation performance. In [15], a SOC-based optimization approach is introduced to minimize overall economic costs, system losses, and line voltage deviations through a penalty function formulation. To investigate the beneficial effect of bi-directional electric-hydrogen conversion on market trading, an optimal scheduling method is proposed in [16] for VPP with reversible solid oxide cells in the electricity market. Given the escalating complexity of dispatchable entities within VPP operations, significant attention has been directed toward enhancing dispatch efficiency [17]. In [18],* *an economic VPP scheduling model considering the load demand under the mechanism of a peak–valley tariff is proposed. To address the uncertainties associated with renewable energy integration, a novel intelligent methodology is proposed in [19] based on fuzzy cloud theory and honeybee mating algorithm.*

*In the source-network-load-storage aspect, the maximum utilization of energy resources is achieved through multiple forms of interaction between power sources, grids, loads and energy storage. In [20], a stochastic economical optimization strategy is proposed for VPPs, addressing scenarios both with and without demand response programs. Based on model predictive control, a multi-stage optimal source-network-load-storage scheduling model is proposed in [21] to ensure the safe and economic operation. In [22], a source-network-load-storage optimization model is introduced, where a point-to-point vehicle charging strategy is implemented. In [23], an integrated source-grid-load-storage monitoring platform based on the micro-grid in State Grid Shanghai Electrical Power Research Institude and blockchain technology. In [24], a novel multi-stage time scale economic dispatch scheme is proposed for VPPs, including a fuzzy optimized day ahead scheduling scheme, an intra-day scheduling scheme combined with Deep Q Network, and an adaptive optimized real-time scheduling scheme. In [25], a bi-layer low-carbon scheduling model is proposed based on node carbon emission intensity. The integration of cloud-edge-end technologies in VPPs is becoming increasingly necessary due to the growing complexity of managing distributed energy resources and ensuring grid stability. VPPs rely on real-time data processing and coordination between various sources of generation, storage, and demand, which can be geographically dispersed and highly dynamic. Together, cloud-edge-end frameworks enhance the flexibility, resilience, and efficiency of VPPs, enabling them to better respond to fluctuations in energy supply and demand, especially in the context of a rapidly evolving electricity-carbon market. However, multi-timescale cloud-edge-end based the VPP source-network-load-storage in the context of electricity-carbon market has not been explored.*

*Real-time pricing (RTP) and time of use (TOU) are two typical pricing mechanisms to facilitate the VPP source-network-load-storage interactions. While RTP reflects the actual cost of electricity based on real-time supply and demand conditions in the market, TOU is set based on predefined time periods, typically distinguishing between peak, off-peak, and mid-peak hours. Though RTP and TOU pricing mechanisms drive more intelligent interactions between VPP source-network-load-storage, the VPP supply–demand behavior is generally ignored. While the end-users can receive financial benefits from the pricing mechanisms, overall system load profile cannot be flattened.”*

Then, to provide a clearer overview of prior research and position our work effectively, we have added a new comparison table. Our method is also included in this table for direct comparison, helping to highlight the novelty and addressed gaps. Following this comment, a comparative table is provided to show the advantages of this work.

*Table 1. DIFFERENCES WITH THE PROPOSED APPROACH*

| *References* | *source-network* | *source-load-storage* | *source-network-load* | *source-network-load-storage* | *pricing* | *Cloud-edge-end* |
| --- | --- | --- | --- | --- | --- | --- |
| *[4][5][9]* | *√* |  |  |  | *TOU* |  |
| *[6][7][8]* | *√* |  |  |  | *RTP* |  |
| *[11]* |  |  | *√* |  | *TOU* | *√* |
| *[12][14]* |  |  | *√* |  | *TOU* |  |
| *[13][15][18]* |  | *√* |  |  | *TOU* |  |
| *[10][16][19]* |  | *√* |  |  | *RTP* |  |
| *[17]* |  |  | *√* |  | *RTP* |  |
| *[20]* |  |  |  | *√* | *RTP* |  |
| *[21]-[25]* |  |  |  | *√* | *TOU* |  |
| *Proposed* |  |  |  | *√* | *interactive RTP* | *√* |

Furthermore, we have revised the contribution section to more clearly and highlight the strengths of the paper. The main contributions of this paper are summarized as follows: 1) While the previous literature is mainly limited to the parts of source–network–load–storage, the inherent system availability and dispatchability from other flexible parts cannot be fully exploited. Moreover, the lack of integrated control strategies often leads to suboptimal system performance and resource utilization. This paper proposes a unified VPP source–network–load–storage management framework, which leverages cloud-edge-end collaborative technologies to achieve the real-time data processing and coordination. Through advanced communication and control mechanisms, heterogeneous energy resources can be managed in a more synchronized manner. This framework enables coordinated and flexible control of distributed generation, distribution networks, EVs, and energy storage systems, thereby reducing both system energy consumption and operational costs; 2) A two-layer cloud–edge–end-based multi-time scale economic management framework for VPP source–network–load–storage is proposed to enable dynamic and hierarchical control of the system. In the first layer, day-ahead economic scheduling of source–network–load–storage resources is performed at the cloud–edge level, ensuring optimal allocation based on forecasted data. In the second layer, a novel real-time supply-demand pricing mechanism is introduced to optimize the scheduling of EV storage and charging stations based on end-side MPC, allowing the system to adapt flexibly to real-time fluctuations. Compared to a single-layer framework, the two-layer model offers enhanced cost-effectiveness and is easier to implement, providing a scalable solution that better accommodates the growing complexity of distributed energy resources; 3) While RTP and TOU pricing mechanisms generally ignore the supply–demand behavior, an interactive real-time pricing mechanism is proposed for EV storage and charging stations. This mechanism dynamically adjusts prices based on real-time supply–demand conditions, aiming to better align economic incentives with system needs. By continuously monitoring system fluctuations, it can more effectively balance load and generation. Moreover, it encourages flexible EV charging behaviors, ultimately enhancing overall grid stability and efficiency. This has been clarified in Section 1.3:

*“1.3. Contribution and paper organization*

*Based on the cloud-edge-end collaborative technology, this paper proposes a multi-time scale VPP source-network-load-storage management framework. This paper makes the following contributions:*

*1) While the previous literature is mainly limited to the parts of source–network–load–storage, the inherent system availability and dispatchability from other flexible parts cannot be fully exploited. This paper proposes a unified VPP source–network–load–storage management framework, which leverages cloud-edge-end collaborative technologies to achieve the real-time data processing and coordination. This framework enables coordinated and flexible control of distributed generation, distribution networks, EVs, and energy storage systems, thereby reducing both system energy consumption and operational costs.*

*2) A two-layer cloud–edge–end-based multi-time scale economic management framework for VPP source–network–load–storage is proposed to enable dynamic and hierarchical control of the system. In the first layer, day-ahead economic scheduling of source–network–load–storage resources is performed at the cloud–edge level.* *In the second layer, a novel real-time supply-demand pricing mechanism is introduced to optimize the scheduling of EV storage and charging stations based on end-side model predictive control (MPC). Compared to a single-layer framework, the two-layer model offers enhanced cost-effectiveness and is easier to implement.*

*3) While RTP and TOU pricing mechanisms generally ignore the supply–demand behavior, an interactive real-time pricing mechanism is proposed for EV storage and charging stations. This mechanism dynamically adjusts prices based on real-time supply–demand conditions, aiming to better align economic incentives with system needs.*

*4) To address the non-convexity inherent in the source-network-load-storage and real-time electricity pricing model, a mixed-integer linear programming (MILP) relaxation is employed. This approach ensures the preservation of convexity and enhances the convergence performance of the optimization process.”*

Comment 4:

*Ensure all figures are presented in high resolution with clarity.*

We appreciate the reviewer’s comment regarding the quality of the figures. In response, we have replaced all relevant figures in the manuscript with high-resolution versions to ensure clarity and readability.

**Figure 3.** Cloud-edge-end-based economical management of VPP source-network-load-storage

**Figure 11.** The cost of carbon trading

Comment 5:

*The authors haven't stated the limitations of their study.*

The authors appreciate the Reviewer for pointing out the need for limitations. In this paper, we propose a cloud-edge-end-based economical management of VPP source-network-load-storage in the context of the electricity-carbon market. This approach aims to optimize the interactions between various energy sources, networks, and storage systems while minimizing operational costs and maximizing energy efficiency. When facing a high-uncertain environment, traditional numerical methods are not enough, and machine learning techniques would be a potential way to enhance decision-making and forecasting accuracy. By leveraging advanced machine learning algorithms, we can better anticipate market fluctuations and improve the overall stability of the energy system. In response, we have added a dedicated paragraph in Section 5 to explicitly outline the key limitations of our work: *“Further research would focus on* *the application of machine learning techniques to VPP source-network-load-storage management under high-uncertain environment.”*

Comment 6:

*Report the statistical analysis in a dedicated section and the significance of results.*

The authors sincerely thank the reviewer for this insightful comment.

In Section 4.2, we conducted a comparative analysis of the proposed two-layer optimization approach against baseline strategies, focusing on key performance indicators such as total operating cost, carbon trading profit, and system flexibility. This has been clarified in Section 4.2: *“Compared to the single-layer optimization strategy, the proposed two-layer approach in this paper achieves a 2.35% reduction in total operating costs and a 2.14% decrease in carbon trading expenses.”*

On the other hand, the proposed two-layer approach can effectively smooth the net load curve and reduce the EV cost. Furthermore, the coordinated scheduling of electric vehicles through the end-side MPC and real-time pricing mechanism effectively reduces EV charging costs. This has been clarified in Section 4.3: *“Compared with the other two electricity pricing strategies, the proposed method results in a reduction of EV-related operational costs by 13.83% and 11.32%, respectively.”*

In addition, based on the reviewer's comments, we have added a description of the power sales and charging stations in Table 2: *“Moreover, the two-layer optimization strategy enhances the utilization efficiency of renewable energy sources, such as photovoltaic and wind power. It should be noted that the purchased electricity from market is assumed to be generated by thermal power unit. By minimizing the dependence on electricity procurement, the proposed scheme significantly reduces carbon emissions during electricity market transactions, thus lowering the overall carbon trading costs. Although the two-layer optimization strategy incurs an additional scheduling expense of $30.88 compared to the single-layer approach, the overall operational cost remains lower. Compared to the single-layer optimization strategy, the proposed two-layer approach in this paper achieves a 2.35% reduction in total operating costs and a 2.14% decrease in carbon trading expenses. However, due to capacity and power output constraints, the contribution of EV storage and charging stations to the overall power supply remains limited and does not exhibit a pronounced effect.”*

Comment 7:

*All the findings of the current work need to be compared and discussed with the results of other researchers*

*finding instead of having a general comparison with other researchers' works. The authors should perform a*

*comparison between the forecasting results. In your discussion section, please link your empirical results with a broader and deeper literature review.*

In Section 4.2, the proposed two-layer scheduling framework is compared with the single-layer approach presented in [4]. Our results demonstrate that the hierarchical coordination strategy achieves superior performance in terms of cost reduction and renewable energy accommodation. Compared to the single-layer optimization strategy, the two-layer approach proposed in this paper achieves a 2.35% reduction in total operating costs and a 2.14% decrease in carbon trading expenses. This has been clarified in Section 4.2:

*“Table 3. Comparison of optimization results between single layer and this solution*

| *Scheme* | *One-layer* | *Two-layer* |
| --- | --- | --- |
| *Buying and selling electricity (kWh)* | *259158.14* | *258552.8926* |
| *Storage and charging stations cost ($)* | *\* | *30.88* |
| *Carbon trading costs ($)* | *2237.31* | *2190.36* |
| *Total cost ($)* | *5011.44* | *4896.29* |

*Table 3 presents a comparative analysis of the operating costs for the source-network-load-storage components of a VPP under two different optimization schemes. In the proposed two-layer scheme, the EV storage and charging stations are capable of storing energy during low-cost periods and subsequently discharging it for sale during peak demand. This reduces the frequency of charging and discharging cycles in the energy storage system, thereby improving economic returns for the VPP.*

*Moreover, the two-layer optimization strategy enhances the utilization efficiency of renewable energy sources, such as photovoltaic and wind power. It should be noted that the purchased electricity from market is assumed to be generated by thermal power unit. By minimizing the dependence on electricity procurement, the proposed scheme significantly reduces carbon emissions during electricity market transactions, thus lowering the overall carbon trading costs. Although the two-layer optimization strategy incurs an additional scheduling expense of $30.88 compared to the single-layer approach, the overall operational cost remains lower. Compared to the single-layer optimization strategy,* *the proposed two-layer approach in this paper achieves a 2.35% reduction in total operating costs and a 2.14% decrease in carbon trading expenses. However, due to capacity and power output constraints, the contribution of EV storage and charging stations to the overall power supply remains limited and does not exhibit a pronounced effect.”*

In Section 4.3, the performance of interactive real-time pricing strategy is benchmarked against both traditional TOU pricing [30] and conventional RTP [31]. As shown in the results and analysis, our proposed pricing mechanism achieves more effective net load smoothing and greater operational cost savings. This has been clarified in Section 4.3:

*“*

***Figure 13****. Net load curves under three pricing schemes*

*Figure 13 illustrates the net load profile under three different pricing mechanisms. It can be found that net load fluctuations are effectively smoothed as energy storage systems and EV charging stations respond to price signals under all three pricing mechanisms. Among them, the proposed pricing mechanism based on real-time net load characteristics exhibits the most favorable performance. EV storage systems and charging stations are observed to charge during periods of system surplus and discharge in response to real-time market prices, thereby improving overall electricity utilization efficiency. Specifically, EV storage and charging stations strategically charge during periods of electricity surplus, typically characterized by lower prices and high renewable generation output. Conversely, during peak demand or when electricity prices surge, these stations discharge stored energy back into the grid, thereby alleviating supply–demand imbalances. This dynamic interaction not only enhances the temporal utilization efficiency of electricity but also contributes to peak shaving, renewable energy accommodation, and overall system flexibility.*

*The proposed scheme demonstrates practical feasibility by effectively mitigating the mismatch between electricity supply and demand, while simultaneously reducing the charging and discharging costs associated with EV storage and charging stations. Compared with the other two electricity pricing strategies, the proposed method results in a reduction of EV-related operational costs by 13.83% and 11.32%, respectively.”*

In Section 4.4, To further highlight the contribution of EV flexibility, we conduct comparative simulations with and without the inclusion of EV storage and charging stations. The results show that systems incorporating EV flexibility achieve superior load distribution and higher clean energy utilization, thereby improving both economic and environmental performance. This has been clarified in Section 4.4:

*“**Without the integration of EV storage and charging stations, the net load exhibits sharp peaks and troughs, reflecting high volatility in power demand and a greater reliance on grid flexibility. Such fluctuations can complicate grid scheduling and increase the need for costly ancillary services. With the proposed method, the net load curve becomes significantly smoother. The EV storage and charging stations effectively act as distributed flexibility assets, absorbing excess generation during low-demand periods and supplying energy during peak demand intervals. This not only reduces the stress on the grid but also enhances system stability and economic performance. These results collectively highlight the critical role of coordinated EV storage and charging strategies in supporting grid flexibility, reducing operational costs, and promoting the integration of renewable energy sources.*

*Figure 15. Net load curves under two scenarios”*

In addition, we've added a new discussion. To address the model's sensitivity to computational parameters, we conducted a sensitivity analysis focusing on the battery degradation cost coefficient, which is closely linked to battery pricing. As shown in the figure 16, we varied the degradation coefficient across a representative range. The results indicate that as the coefficient increases, the system’s total cost correspondingly rises. Notably, when the coefficient reaches 0.06, the battery ceases to operate due to economic infeasibility, and the total cost plateaus thereafter. This behaviour confirms that the model responds reasonably to parameter changes and reflects realistic system decision-making under different economic assumptions. This has been clarified in Section 4.5:

*“4.5.* *Sensitivity analysis on battery degradation cost coefficient*

*Battery degradation cost is a key economic parameter that directly affects the decision-making of energy storage. It reflects both the replacement cost of batteries and the marginal cost of usage. To evaluate the sensitivity to this parameter, we conduct a series of simulations by varying the battery degradation cost coefficient from 0.01 to 0.07.*

*With the increase of battery degradation cost coefficient from 0.001 to 0.06, the total system operating cost gradually rises. The battery is used in a limited manner to circumvent the high battery degradation costs, resulting in increased reliance on grid electricity or alternative resources. With the increase of battery degradation cost reaches 0.06, the degradation cost becomes comparable to or higher than the benefit of battery participation, resulting in avoiding battery operation to minimize system cost. Beyond this threshold, further increases in the degradation cost coefficient do not impact the total cost, as the battery is no longer utilized.*

***Figure 16.*** *Sensitivity analysis of system operating cost with respect to battery degradation cost coefficient”*

Comment 8:

*Conclusions: This section is too general, please add some information about the value of obtained parameters of tested the series as well as information about the novelty of the proposed technology.*

As suggested, the conclusion of the paper is modified to highlight the information by referring to numerical results in case study. This has been clarified in Section 5:

*“**This paper presents a cloud–edge–end multi-time scale economic management framework for VPP source-network-load-storage under the electricity–carbon market environment. In the first layer, a day-ahead source-network-load-storage optimization model is formulated to minimize the overall operational cost. In the second layer, an MPC-based real-time strategy is employed for the scheduling of EV storage and charging stations, aiming to mitigate the uncertainty and intermittency associated with RESs. Comparative analysis demonstrates that:*

*1) By optimizing the interaction among various system components, the coordination of source-network-load-storage enhances the dispatchability of the system across both spatial and temporal domains, thereby reducing at most 13.83% EV cost.*

*2) The deployment of EV storage and charging stations equipped with real-time pricing mechanisms enables dynamic load balancing and more efficient resource utilization, which leads to a 2.35% reduction in overall operational costs and a 2.14% reduction in carbon trading expenditures.*

*3) The proposed two-layer optimization approach outperforms other methods in terms of energy savings and net load management, demonstrating significant potential for application in systems with abundant renewable energy sources.”*

Reviewer 2

Comment 1:

Add some of the most important quantitative results to the abstract. Focus on the advantages of the proposed method with respect to the obtained results.

As suggested, the Abstract section is enhanced with some numerical results added. Simulations results show that the system operating cost can be reduced by at most 2.35% with a higher flexibility of electricity market operations. This has been clarified in Abstract: *“Under the imperative of achieving dual-carbon goals, the number of distributed energy resources are gradually increasing, thereby amplifying the challenges to grid stability and power balance. Consequently, there is an urgent need to leverage the potential of source-network-load-storage for enhanced power regulation and control. This paper proposes a cloud-edge-end-based multi-time scale economical management of virtual power plant (VPP) source-network-load-storage in the context of electricity-carbon market. In the first layer, a cloud-edge scheduling approach is used to optimize the source-network-load-storage system of the VPP over a long time horizon, aiming to maximize economic benefits. In the second layer, a novel real-time pricing mechanism is employed to effectively manage and regulate the electric vehicle (EV) storage and charging stations. After obtaining the economic management parameters from the previous layer, the second layer employs a real-time scheduling approach based on end-side model predictive control (MPC), to address multi-energy supply-demand fluctuations. To achieve efficient solution, the original two-layer optimization problem is reformulated using mixed-integer linear programming (MILP). Comparative analyses have demonstrated the superior economic and practical performance of the pro-posed two-layer optimization approach. Simulation results indicate that the total operating cost of the system can be reduced by 2.35%, with a higher flexibility of electricity market operations.”*

Comment 2:

*Most of the ideas written were already described in many literatures. The Authors tried to compile it but lack*

*of the enhancement of the interrelation analysis between the references. It is advised that the authors give a deeper analysis on how these ideas become more applicative strategies so that they can contribute to the next step of implementation.*

As suggested, we have updated our references with the latest researches, including the suggested references and the following references:

[11]	Alsokhiry, F.; Annuk, A.; Mohamed, M.A.; Marinho, M. An innovative cloud-fog-based smart grid scheme for efficient resource utilization. Sensors. **2023,** 23, 1752.

[12]	Li, P.; Zheng M. Multi-objective optimal operation of hybrid AC/DC microgrid considering source-network-load coordination, Modern Power Systems and Clean Energy, **2023**, 7, 1229.

[16]	Wang, Q.; Leng, Z.; Tan, H.; Mohamed A. Mohamed. Optimal scheduling of virtual power plants with reversible solid oxide cells in the electricity market, Renewable Energy. **2024**, 237, 121759.

[18]	He, Y.; Wu, X.; Sun, K.; Du, X.; Wang, H.; Zhao, J. Economic optimization scheduling based on load demand in microgrids considering source network load storage. Electronics. **2023**, 12, 2721.

[19]	Mohamed A.; Heba M.; Mohammed A.; Mohamed Sharaf.; Ahmed T. A novel fuzzy cloud stochastic framework for energy management of renewable microgrids based on maximum deployment of electric vehicles, International Journal of Electrical Power & Energy Systems. **2021**, 129, 106845.

[25]	Liu, X. Research on bi-layer low carbon scheduling strategy for source-load collaborative optimization based on node carbon emission intensity. Energy Science & Engineering. **2024**; 12: 713-735.

*Table 1. DIFFERENCES WITH THE PROPOSED APPROACH*

| *References* | *source-network* | *source-load-storage* | *source-network-load* | *source-network-load-storage* | *pricing* | *Cloud-edge-end* |
| --- | --- | --- | --- | --- | --- | --- |
| *[4][5][9]* | *√* |  |  |  | *TOU* |  |
| *[6][7][8]* | *√* |  |  |  | *RTP* |  |
| *[11]* |  |  | *√* |  | *TOU* | *√* |
| *[12][14]* |  |  | *√* |  | *TOU* |  |
| *[13][15][18]* |  | *√* |  |  | *TOU* |  |
| *[10][16][19]* |  | *√* |  |  | *RTP* |  |
| *[17]* |  |  | *√* |  | *RTP* |  |
| *[20]* |  |  |  | *√* | *RTP* |  |
| *[21]-[25]* |  |  |  | *√* | *TOU* |  |
| *Proposed* |  |  |  | *√* | *interactive RTP* | *√* |

And in the literature review we have added the following summary: *“The integration of cloud-edge-end technologies in VPPs is becoming increasingly necessary due to the growing complexity of managing distributed energy resources and ensuring grid stability. VPPs rely on real-time data processing and coordination between various sources of generation, storage, and demand, which can be geographically dispersed and highly dynamic. Together, cloud-edge-end frameworks enhance the flexibility, resilience, and efficiency of VPPs, enabling them to better respond to fluctuations in energy supply and demand, especially in the context of a rapidly evolving electricity-carbon market. However, multi-timescale cloud-edge-end based the VPP source-network-load-storage in the context of electricity-carbon market has not been explored.*

*Real-time pricing (RTP) and time of use (TOU) are two typical pricing mechanisms to facilitate the VPP source-network-load-storage interactions. While RTP reflects the actual cost of electricity based on real-time supply and demand conditions in the market, TOU is set based on predefined time periods, typically distinguishing between peak, off-peak, and mid-peak hours. Though RTP and TOU pricing mechanisms drive more intelligent interactions between VPP source-network-load-storage, the VPP supply–demand behavior is generally ignored. While the end-users can receive financial benefits from the pricing mechanisms, overall system load profile cannot be flattened.”*

Comment 3:

*The literature review could be greatly improved. The authors first need to make comparisons of the references and then draw the motivation of the paper. Neither the comparison of references and this work nor the corresponding conclusion is made in the paper. Thus, it is difficult for me to know the novelty and advantages of this paper over other works. To improve this part, the following relevant recent publications can*

*be discussed: Optimal scheduling of virtual power plants with reversible solid oxide cells in the electricity market; An Innovative Cloud-Fog-Based Smart Grid Scheme for Efficient Resource Utilization; A novel fuzzy cloud stochastic framework for energy management of renewable microgrids based on maximum deployment of electric vehicles.*

The authors totally agree with the Reviewer for this suggestion. In the revised paper, more recently published

works related have been cited, as follows:

[11]	Alsokhiry, F.; Annuk, A.; Mohamed, M.A.; Marinho, M. An innovative cloud-fog-based smart grid scheme for efficient resource utilization. Sensors. **2023,** 23, 1752.

[12]	Li, P.; Zheng M. Multi-objective optimal operation of hybrid AC/DC microgrid considering source-network-load coordination, Modern Power Systems and Clean Energy, **2023**, 7, 1229.

[16]	Wang, Q.; Leng, Z.; Tan, H.; Mohamed A. Mohamed. Optimal scheduling of virtual power plants with reversible solid oxide cells in the electricity market, Renewable Energy. **2024**, 237, 121759.

[18]	He, Y.; Wu, X.; Sun, K.; Du, X.; Wang, H.; Zhao, J. Economic optimization scheduling based on load demand in microgrids considering source network load storage. Electronics. **2023**, 12, 2721.

[19]	Mohamed A.; Heba M.; Mohammed A.; Mohamed Sharaf.; Ahmed T. A novel fuzzy cloud stochastic framework for energy management of renewable microgrids based on maximum deployment of electric vehicles, International Journal of Electrical Power & Energy Systems. **2021**, 129, 106845.

[25]	Liu, X. Research on bi-layer low carbon scheduling strategy for source-load collaborative optimization based on node carbon emission intensity. Energy Science & Engineering. **2024**; 12: 713-735.

Following this comment, the introduction presents a comprehensive examination of VPP source–network–load–storage co-optimization to underscore the novelty of the proposed framework. The review not only provides a thorough background based on state-of-the-art research but also critically identifies the limitations of existing approaches. Until now, extensive researches in [4]-[19] on VPP is mainly limited to parts of source–network–load–storage, including source–storage, source–load–storage, source–network, etc. Though VPP source–network–load–storage is investigated in [20]-[25], multi-timescale cloud-edge-end based the VPP source-network-load-storage in the context of electricity-carbon market has not been explored. Cloud-edge-end frameworks enhance the flexibility, resilience, and efficiency of VPPs, enabling them to better respond to fluctuations in energy supply and demand, especially in the context of a rapidly evolving electricity-carbon market. Real-time pricing (RTP) and time of use (TOU) are two typical pricing mechanisms to facilitate the VPP source-network-load-storage interactions. Though RTP and TOU pricing mechanisms drive more intelligent interactions between VPP source-network-load-storage, the VPP supply–demand behavior is generally ignored. While the end-users can receive financial benefits from the pricing mechanisms, overall system load profile cannot be flattened. These research gaps and associated challenges are further elaborated in Section 1.2:

*“1.2. Literature review*

*Until now, extensive researches on VPP is mainly limited to parts of source–network–load–storage, including source–storage, source–load–storage, source–network, etc. In the VPP source-network aspect, studies mainly focus on the coordination between distributed energy sources and the power network to enhance system stability and flexibility. In [4],* *advanced control technologies are employed to optimize the interaction between distributed generation units and the grid, improving voltage regulation, frequency support, and fault management capabilities. The complementary characteristics of various RESs are highlighted in [5], since their diverse generation profiles can compensate for each other's intermittency. In [6–7], an enhanced real-time economic scheduling algorithm is developed to improve the accuracy of output prediction for distributed energy resources. In [8], a resilient restoration method is proposed for VPP power-traffic network, which co-optimizes the deployment of mobile power sources and network reconfiguration. In [9], a multi-energy coordination framework is proposed to enhance the VPP resilience against large-scale disturbances, where consumer-side biogas-solar-wind multi-energy VPPs are topologized by network reconfiguration. In [10], the integration of multi-source coordinated scheduling within VPPs is analyzed, with particular emphasis on its performance in ancillary service markets.*

*In the VPP source-load-storage and source-network-load aspects, demand response and load shedding strategies [11] are extensively utilized to enhance system flexibility by dynamically adjusting energy consumption behaviors and patterns. In [12], a multi-objective source-network-load coordinated model is proposed and solved an improved memetic algorithm, considering the adjustment of load demand curves. To exploit the inherent similarities between RESs and load profiles, a two-layer VPP optimization framework is proposed in [13] to jointly coordinate ESS and industrial loads. In [14], an economic scheduling optimization model is developed, explicitly considering the effects of temperature variations on PV generation performance. In [15], a SOC-based optimization approach is introduced to minimize overall economic costs, system losses, and line voltage deviations through a penalty function formulation. To investigate the beneficial effect of bi-directional electric-hydrogen conversion on market trading, an optimal scheduling method is proposed in [16] for VPP with reversible solid oxide cells in the electricity market. Given the escalating complexity of dispatchable entities within VPP operations, significant attention has been directed toward enhancing dispatch efficiency [17]. In [18],* *an economic VPP scheduling model considering the load demand under the mechanism of a peak–valley tariff is proposed. To address the uncertainties associated with renewable energy integration, a novel intelligent methodology is proposed in [19] based on fuzzy cloud theory and honeybee mating algorithm.*

*In the source-network-load-storage aspect, the maximum utilization of energy resources is achieved through multiple forms of interaction between power sources, grids, loads and energy storage. In [20], a stochastic economical optimization strategy is proposed for VPPs, addressing scenarios both with and without demand response programs. Based on model predictive control, a multi-stage optimal source-network-load-storage scheduling model is proposed in [21] to ensure the safe and economic operation. In [22], a source-network-load-storage optimization model is introduced, where a point-to-point vehicle charging strategy is implemented. In [23], an integrated source-grid-load-storage monitoring platform based on the micro-grid in State Grid Shanghai Electrical Power Research Institude and blockchain technology. In [24], a novel multi-stage time scale economic dispatch scheme is proposed for VPPs, including a fuzzy optimized day ahead scheduling scheme, an intra-day scheduling scheme combined with Deep Q Network, and an adaptive optimized real-time scheduling scheme. In [25], a bi-layer low-carbon scheduling model is proposed based on node carbon emission intensity. The integration of cloud-edge-end technologies in VPPs is becoming increasingly necessary due to the growing complexity of managing distributed energy resources and ensuring grid stability. VPPs rely on real-time data processing and coordination between various sources of generation, storage, and demand, which can be geographically dispersed and highly dynamic. Together, cloud-edge-end frameworks enhance the flexibility, resilience, and efficiency of VPPs, enabling them to better respond to fluctuations in energy supply and demand, especially in the context of a rapidly evolving electricity-carbon market. However, multi-timescale cloud-edge-end based the VPP source-network-load-storage in the context of electricity-carbon market has not been explored.*

*Real-time pricing (RTP) and time of use (TOU) are two typical pricing mechanisms to facilitate the VPP source-network-load-storage interactions. While RTP reflects the actual cost of electricity based on real-time supply and demand conditions in the market, TOU is set based on predefined time periods, typically distinguishing between peak, off-peak, and mid-peak hours. Though RTP and TOU pricing mechanisms drive more intelligent interactions between VPP source-network-load-storage, the VPP supply–demand behavior is generally ignored. While the end-users can receive financial benefits from the pricing mechanisms, overall system load profile cannot be flattened.”*

Comment 4:

*The relevant discussion and content of the figures and tables in the text are not enough.*

We thank the reviewer for pointing out the insufficient discussion of figures and tables in the manuscript. In response, we have revised the manuscript extensively to enhance the integration of figures and tables into the main body of the text. Specifically:

*“Table 2 presents a comparative analysis of the operating costs for the source-network-load-storage components of a VPP under two different optimization schemes. In the proposed two-layer scheme, the EV storage and charging stations are capable of storing energy during low-cost periods and subsequently discharging it for sale during peak demand. This reduces the frequency of charging and discharging cycles in the energy storage system, thereby improving economic returns for the VPP.*

*Moreover, the two-layer optimization strategy enhances the utilization efficiency of renewable energy sources, such as photovoltaic and wind power. It should be noted that the purchased electricity from market is assumed to be generated by thermal power unit. By minimizing the dependence on electricity procurement, the proposed scheme significantly reduces carbon emissions during electricity market transactions, thus lowering the overall carbon trading costs. Although the two-layer optimization strategy incurs an additional scheduling expense of $30.88 compared to the single-layer approach, the overall operational cost remains lower. Compared to the single-layer optimization strategy, the proposed two-layer approach in this paper achieves a 2.35% reduction in total operating costs and a 2.14% decrease in carbon trading expenses. However, due to capacity and power output constraints, the contribution of EV storage and charging stations to the overall power supply remains limited and does not exhibit a pronounced effect.”*

*“Figure 12 illustrates the net load profile under three different pricing mechanisms. It can be found that net load fluctuations are effectively smoothed as energy storage systems and EV charging stations respond to price signals under all three pricing mechanisms. Among them, the proposed pricing mechanism based on real-time net load characteristics exhibits the most favourable performance. EV storage systems and charging stations are observed to charge during periods of system surplus and discharge in response to real-time market prices, thereby improving overall electricity utilization efficiency. Specifically, EV storage and charging stations strategically charge during periods of electricity surplus, typically characterized by lower prices and high renewable generation output. Conversely, during peak demand or when electricity prices surge, these stations discharge stored energy back into the grid, thereby alleviating supply–demand imbalances. This dynamic interaction not only enhances the temporal utilization efficiency of electricity but also contributes to peak shaving, renewable energy accommodation, and overall system flexibility.*

*The proposed scheme demonstrates practical feasibility by effectively mitigating the mismatch between electricity supply and demand, while simultaneously reducing the charging and discharging costs associated with EV storage and charging stations. Compared with the other two electricity pricing strategies, the proposed method results in a reduction of EV-related operational costs by 13.83% and 11.32%, respectively.”*

Comment 5:

*There are several up-to-date approaches for the idea. Authors should look for these approaches, compare the results and prove their idea.*

In Section 4.2, the proposed two-layer scheduling framework is compared with the single-layer approach presented in [4]. Our results demonstrate that the hierarchical coordination strategy achieves superior performance in terms of cost reduction and renewable energy accommodation. Compared to the single-layer optimization strategy, the two-layer approach proposed in this paper achieves a 2.35% reduction in total operating costs and a 2.14% decrease in carbon trading expenses. This has been clarified in Section 4.2:

*“****Table 3****. Comparison of optimization results between single layer and this solution*

| *Scheme* | *One-layer* | *Two-layer* |
| --- | --- | --- |
| *Buying and selling electricity (kWh)* | *259158.14* | *258552.8926* |
| *Storage and charging stations cost ($)* | *\* | *30.88* |
| *Carbon trading costs ($)* | *2237.31* | *2190.36* |
| *Total cost ($)* | *5011.44* | *4896.29* |

*Table 3 presents a comparative analysis of the operating costs for the source-network-load-storage components of a VPP under two different optimization schemes. In the proposed two-layer scheme, the EV storage and charging stations are capable of storing energy during low-cost periods and subsequently discharging it for sale during peak demand. This reduces the frequency of charging and discharging cycles in the energy storage system, thereby improving economic returns for the VPP.*

*Moreover, the two-layer optimization strategy enhances the utilization efficiency of renewable energy sources, such as photovoltaic and wind power. It should be noted that the purchased electricity from market is assumed to be generated by thermal power unit. By minimizing the dependence on electricity procurement, the proposed scheme significantly reduces carbon emissions during electricity market transactions, thus lowering the overall carbon trading costs. Although the two-layer optimization strategy incurs an additional scheduling expense of $30.88 compared to the single-layer approach, the overall operational cost remains lower. Compared to the single-layer optimization strategy, the proposed two-layer approach in this paper achieves a 2.35% reduction in total operating costs and a 2.14% decrease in carbon trading expenses. However, due to capacity and power output constraints, the contribution of EV storage and charging stations to the overall power supply remains limited and does not exhibit a pronounced effect.”*

In Section 4.3, the performance of interactive real-time pricing strategy is benchmarked against both traditional TOU pricing [30] and conventional RTP [31]. As shown in the results and analysis, our proposed pricing mechanism achieves more effective net load smoothing and greater operational cost savings. This has been clarified in Section 4.2:

*“*

***Figure 13****. Net load curves under three pricing schemes*

*Figure 13 illustrates the net load profile under three different pricing mechanisms. It can be found that net load fluctuations are effectively smoothed as energy storage systems and EV charging stations respond to price signals under all three pricing mechanisms. Among them, the proposed pricing mechanism based on real-time net load characteristics exhibits the most favourable performance. EV storage systems and charging stations are observed to charge during periods of system surplus and discharge in response to real-time market prices, thereby improving overall electricity utilization efficiency. Specifically, EV storage and charging stations strategically charge during periods of electricity surplus, typically characterized by lower prices and high renewable generation output. Conversely, during peak demand or when electricity prices surge, these stations discharge stored energy back into the grid, thereby alleviating supply–demand imbalances. This dynamic interaction not only enhances the temporal utilization efficiency of electricity but also contributes to peak shaving, renewable energy accommodation, and overall system flexibility.*

*The proposed scheme demonstrates practical feasibility by effectively mitigating the mismatch between electricity supply and demand, while simultaneously reducing the charging and discharging costs associated with EV storage and charging stations. Compared with the other two electricity pricing strategies, the proposed method results in a reduction of EV-related operational costs by 13.83% and 11.32%, respectively.”*

In Section 4.4, To further highlight the contribution of EV flexibility, we conduct comparative simulations with and without the inclusion of EV storage and charging stations. The results show that systems incorporating EV flexibility achieve superior load distribution and higher clean energy utilization, thereby improving both economic and environmental performance. This has been clarified in Section 4.4:

*“Without the integration of EV storage and charging stations, the net load exhibits sharp peaks and troughs, reflecting high volatility in power demand and a greater reliance on grid flexibility. Such fluctuations can complicate grid scheduling and increase the need for costly ancillary services. With the proposed method, the net load curve becomes significantly smoother. The EV storage and charging stations effectively act as distributed flexibility assets, absorbing excess generation during low-demand periods and supplying energy during peak demand intervals. This not only reduces the stress on the grid but also enhances system stability and economic performance. These results collectively highlight the critical role of coordinated EV storage and charging strategies in supporting grid flexibility, reducing operational costs, and promoting the integration of renewable energy sources.*

***Figure 15.*** *Net load curves under two scenarios”*

Comment 6:

*Author must justify the choice of solution method. How do authors guarantee optimality of the obtained solutions?*

The authors sincerely thank the Reviewer for the thoughtful and constructive comment. In our study, the entire two-layer optimization model is initially formulated as a MINLP problem. After applying piecewise linearization to equations (2a) and (2c), the remaining nonlinearities are limited to the bilinear terms present in equation (9a). To address these, we adopt the McCormick envelope method [28], which introduces auxiliary variables to replace the bilinear terms and imposes linear envelope constraints to bound these new variables. Through this reformulation, the optimization model is transformed into a MILP problem. Furthermore, it has been demonstrated that the variable $P_{i,t}^{dis}$ $P_{i,t}^{\text{ch}}$ = 0 always holds when the charging/discharging efficiency $\eta_{i,t}^{ch}\eta_{i,t}^{dis}$ <1, allowing for the relaxation of certain binary variables associated with the energy storage system. That is to say, the binary variables related to storage operation can be reasonably neglected, enabling the final model to be approximated as a LP problem. As a result, the final optimization model is convex, and the solution obtained by CPLEX corresponds to the global optimum. We have revised the manuscript accordingly to clarify this model and solution process：

*“Figure 3 is two-layer optimization flowchart of the proposed VPP source-network-load-storage.* *The entire two-layer optimization model in this study is formulated as a MINLP problem, which cannot be easily solved using off-the-shelf solvers directly. In such cases, the original MINLP model is first linearized using the McCormick envelope method [28], and then can be equivalent to an LP model by removing binary variables [29]. This results in a convex optimization problem with a unique global optimum, ensuring efficient and reliable scheduling.”*

Comment 7:

*What is the main driver of optimizing cost? Electricity price? Please add figure with electricity price data.*

In our study, the main driver of the proposed two-layer optimization is the minimization of the total cost of source-network-load-storage coordination. The total cost includes multiple components, such as: battery degradation expenses, costs due to wind curtailment, costs due to solar curtailment, and costs stemming from load shedding, carbon trading cost under the electricity-carbon market. Electricity price plays an important role as an economic signal that affects market-related components, but it is only one part of the objective function.

*“The objective function for the first level can be formulated as follows:*

$F_{u}:\min\left(\sum_{t_{l}\in\left\{1,...,T_{u}\right\}} \left(C_{B}\left(t_{u}\right)+C_{G}\left(t_{u}\right)+C_{n}\left(t_{u}\right)\right)+C_{CO2}\right)$ (8a)

 (8b)

 (8c)

 (8d)

 (8e)”

**Figure 4** Grid electricity price

Figure 4 illustrates the 24-hour electricity price profile. The electricity pricing data in this study is adopted from [27].

Comment 8:

*The authors must explain how their approach can handle the uncertainties*.

In the revised manuscript, we have clarified that renewable energy uncertainty is explicitly addressed through a scenario-based modeling approach. Specifically, we adopt the Monte Carlo scenario generation method, as referenced in [27], to capture the stochastic behavior of renewable energy outputs. This technique allows us to model the variability and randomness inherent in renewable sources by generating a representative set of possible future outcomes based on probabilistic distributions. These generated scenarios are incorporated into both layers of the proposed optimization framework: In the first layer, scenarios are used to support long-term cloud-edge scheduling decisions under different possible future states of the renewable generation. In the second layer, real-time control MPC adapts to unfolding uncertainties by re-optimizing based on updated states, as discussed previously. This hybrid uses of offline Monte Carlo scenario analysis and online MPC feedback control ensure that both forecast-based uncertainty and real-time variability are effectively managed. The treatment of uncertainty is now more clearly described in Section 3.5 of the revised manuscript. *“**The uncertainty associated with renewable energy generation is modeled using a Monte Carlo scenario-based approach [27]. A set of representative scenarios is generated to reflect the stochastic nature of renewable resources, considering historical data and probabilistic distributions. These scenarios are incorporated into the upper-layer optimization to ensure robust scheduling decisions under uncertain conditions. Additionally, the end-side control layer employs MPC, which further mitigates real-time uncertainty by continuously updating the control strategy based on the latest system information.”*

Comment 9:

*What's the sensitivity of the model with the change of computational parameters?*

The authors sincerely thank the Reviewer for the thoughtful comment. To address the sensitivity to computational parameters, we conducted a sensitivity analysis of the battery degradation cost coefficient, which is closely linked to battery capital price. As shown in the figure 16, we varied the degradation coefficient across a representative range. As the coefficient increases, the total system cost correspondingly rises. When the coefficient reaches 0.06, the battery ceases to operate due to economic infeasibility, and the total cost reaches a plateau thereafter. This has been clarified in Section 4.5:

*“4.5. Sensitivity analysis on battery degradation cost*

*Battery degradation cost is a key economic parameter that directly affects the decision-making of energy storage. It reflects both the replacement cost of batteries and the marginal cost of usage. To evaluate the sensitivity to this parameter, we conduct a series of simulations by varying the battery degradation cost coefficient from 0.01 to 0.07.*

*With the increase of battery degradation cost coefficient from 0.001 to 0.06, the total system operating cost gradually rises. The battery is used in a limited manner to circumvent the high battery degradation costs, resulting in increased reliance on grid electricity or alternative resources. With the increase of battery degradation cost reaches 0.06, the degradation cost becomes comparable to or higher than the benefit of battery participation, resulting in avoiding battery operation to minimize system cost. Beyond this threshold, further increases in the degradation cost coefficient do not impact the total cost, as the battery is no longer utilized.*

***Figure 16.*** *Sensitivity analysis of system operating cost with respect to battery degradation cost coefficient*

*”*

**_<END OF AUTHOR RESPONSE FILE>_**
