# Supplementary material for: Economical management of virtual power plant source-network-load-storage in the context of electricity-carbon market
Source: PLoS One. 2026 Jan 7;21(1):e0338321. doi: 10.1371/journal.pone.0338321 (PMC12779080; doi:10.1371/journal.pone.0338321)
Supplement: Attachment — Submitted filename: Response_to_Reviewers_auresp_2.docx [file pone.0338321.s002.docx]

**Authors’ response to the review of paper PONE-D-25-05045**

**Paper title:**	Economical Management of Virtual Power Plant Source-network-load-storage in the Context of Electricity-Carbon Market

**Authors:**	Panhong Zhang and Yilu Zheng

**Manuscript number:	PONE-D-25-05045**

**Submission 1:**	Jan 29 2025

**Revision 1 request:**	Mar 31 2025

**Submission 2:**	May 15 2025

**Revision 2 request:**	Jun 23 2025

**Submission 3:**	Jun 27 2025

Editor’s Comments

*Thank you for submitting your manuscript to* *PLOS ONE. After careful consideration, we feel that it has merit but does not fully meet PLOS ONE’s publication criteria as it currently stands. Therefore, we invite you to submit a revised version of the manuscript that addresses the points raised during the review process.*

The Reviewers have meticulously examined our manuscript and presented us with a very constructive set of comments and requests, which we believe have improved the strength of our manuscript and the presentation of our work significantly. In the following pages, we address each and every comment. Each section discusses one comment, and refers to the particular point of our revised manuscript that has been amended to accommodate this comment.

We have endeavoured to take all the remarks and requests of the Reviewers strongly into account, and have addressed all comments analytically and very carefully, no matter how minor or major they were. We hope our revision is now up to the standards of **PLOS ONE** and if requested, we would be glad to address any further issues in subsequent revisions.

Additional Editor Comments

Comment 1:

*1)The introduction section still needs improvements.*

*Add more details about the problem being investigated in the introduction section.*

*Descuss how this research adreeses the problem (research methodology).*

*Remove the literature review (Sec. 1.2) and put it in a separate section.*

*Merge Sec. 1.1. and 1.3. Moreover add the final paragraph that describes the whole organization and flow of the paper at the end.*

The Reviewers sincerely appreciate your detailed and constructive comments. Below, we address each of your suggestions and describe the corresponding revisions made in the manuscript:

First of all, in the revised manuscript, we have enhanced the Introduction section by providing a more detailed description of the problem being investigated. Specifically, we have clearly articulated the challenges associated with achieving coordinated economic scheduling of source–network–load–storage systems under the electricity–carbon market framework. This has been clarified in Section 1:

*“Against the backdrop of profound energy structure adjustment, the share of renewable energy in the power system has been growing rapidly. The intermittency and uncertainty of new energy sources present significant challenges to the safe and stable operation of the power grid. A virtual power plant (VPP) represents a centralized network comprising decentralized energy resources, including photovoltaic (PV), wind turbines (WT), energy storage systems (ESS), and flexible demand-side loads. These resources are aggregated and controlled uniformly to mimic the operation of a single, unified power plant. The VPP concept emerged in response to the widespread adoption of renewable energy sources (RESs) and the pressing demand for flexible, interactive, and efficient grid systems [1]. Traditional power grids, often reliant on large, centralized fossil fuel-based power plants, face challenges in handling the RES volatility [2]. VPPs provide a solution by facilitating enhanced coordination of source-network-load-storage systems., making it possible to balance supply and demand.*

*The motivation behind VPPs is to maximize the use of RESs, enhance grid stability, and achieve the dual carbon goal [3]. By aggregating small-scale energy resources, VPPs are capable of offering network services, including load balancing and frequency regulation., and peak shaving. Moreover, VPPs can improve the economics of renewable energy generation, making it more cost-effective for consumers and reducing reliance on fossil fuels. VPPs also support the transition to a decentralized new energy system, empowering consumers to become active participants in electricity-carbon markets. Therefore, the optimal scheduling of VPPs with source-network-load-storage collab-oration has become an important topic.”*

Then, we have clarified how the proposed methodology addresses the core research problem. The main challenge lies in the coordinated economic optimization of VPP source-network-load-storage under market-driven conditions. To address this, we develop a two-layer optimization framework: the upper layer performs long-term scheduling based on cloud-edge collaboration, integrating electricity-carbon market prices to guide the VPP source-network-load-storage dispatch for overall economic efficiency; the lower layer manages real-time fluctuations through an end-side model predictive control (MPC) scheme, guided by a transaction-based real-time pricing mechanism. This enables responsive EV storage and charging stations charging behaviour and flexible demand regulation. To ensure computational tractability, the model is reformulated as a MILP, and further relaxed into an LP, guaranteeing a globally optimal and unique solution. This hierarchical approach directly tackles the challenges of distributed coordination, market coupling, and real-time responsiveness, as detailed in the revised Section 1.

*“To address economical management coordination problem of VPP in the context of electricity-carbon market, this paper proposes a multi-time scale VPP source-network-load-storage management framework, based on the cloud-edge-end collaborative technology. The upper layer utilizes cloud–edge collaborative scheduling to optimize long-term VPP operations, incorporating electricity–carbon market dynamics for overall economic efficiency. The lower layer applies an end-side model predictive control (MPC) strategy to manage EV storage and charging stations charging behavior and balance short-term supply and demand, driven by a transaction-based real-time pricing mechanism. This hierarchical approach enables scalable, adaptive, and economically efficient VPP scheduling.”*

Furthermore, we have restructured the Introduction by merging the former Sections 1.1 and 1.3 to provide a more concise and coherent problem statement and motivation. In the revised Introduction, we have added a final paragraph that outlines the overall organization and flow of the paper, allowing readers to better follow the structure and contributions of the work. This has been clarified in Section 1:

*“The rest of this paper is organized as follows. In Section 2, this paper introduces some research advances of VPP source-network-load-storage collaboration. In Section 3, an operation framework for VPP source-network-load-storage based on cloud-edge-end technology is outlined. In Section 4, the VPP source-network-load-storage scheduling model is established. In Section 5, the proposed VPP source-network-load-storage is subjected to simulation analysis. Finally, several highlights are concluded of this paper in Section 6.”*

Finally, the literature review (previously Section 1.2) has now been moved to a new standalone section titled “Literature review” (Section 2) to enhance clarity and logical organization.

Comment 2:

*Proofread the article to ensure appropriate use of English grammar, tenses, and punctuation. Longer sentences should be broken out into smaller ones. There are also some linguistic issues that should be corrected. The use of article "the" is redundant and somewhere missing.*

We appreciate the reviewer’s insightful comment. We carefully proofread the entire manuscript to improve the clarity and correctness of English usage. Specifically: Grammar, punctuation, and verb tenses have been thoroughly reviewed and corrected. Overly long or complex sentences have been split into shorter, more readable segments. The usage of the definite article “the” has been revised throughout the paper to correct both redundancies and omissions. We also refined technical phrasing and polished expressions to meet academic writing standards. We believe these revisions significantly improve the readability and overall presentation of the manuscript.

Comment 3:

*The notation table can be put in Sec 2 (problem formulation).*

Thank you for the helpful suggestion. Accordingly, we have moved the notation table to Section 3 (Problem Formulation) to improve clarity and consistency. Placing the notation table alongside the mathematical modelling helps readers better understand the variables and symbols used throughout the formulation.

Comment 4:

*Improve the organization of the paper.*

Thank you for your constructive suggestion regarding the paper’s organization. In the revised manuscript, we have improved the overall structure to enhance clarity and logical flow. Specifically: We have merged the original Sections 1.2 and 1.3 into a more concise and focused introduction. The revised Introduction now includes a streamlined motivation, a clearer problem statement, and a bullet-point summary of the main contributions. We have moved the notation table into the original Section 2 (now Section 3) to support immediate understanding of the mathematical model and enhance the readability of the problem formulation. These changes improve the coherence between sections and make the manuscript more accessible to readers.

Last but not least, this manuscript has been thoroughly revised with all Reviewers’ comments, suggestions and corrections incorporated. Compared with the previous submission, throughout the manuscript numerous phrases and sentences have been rewritten to ensure the coherence and consistency of the proposed methodology. We also would like to report that both the technical and grammatical presentation of the revised paper has been thoroughly polished to provide a well-organized paper for readers.

**_<END OF AUTHOR RESPONSE FILE>_**
